# Long-term impact of the adoption of bedaquiline-containing regimens on the burden of drug-resistant tuberculosis in China

**DOI:** 10.1186/s12879-020-4795-4

**Published:** 2020-02-10

**Authors:** Abela Mpobela Agnarson, Xiao Chun Wang, Ravi Potluri, Hitesh Bhandari, Amit Dhir, Chrispin Kambili, Laurent Metz

**Affiliations:** 1 Johnson & Johnson Services, Inc., New Brunswick, NJ USA; 2 Xian Janssen Pharmaceutical Ltd., Beijing, China; 3 SmartAnalyst Inc., New York, NY USA; 4 SmartAnalyst India Pvt. Ltd., Gurugram, India; 5 Johnson & Johnson Services, Inc., Raritan, NJ USA

**Keywords:** Drug-resistant tuberculosis, Bedaquiline, China, Disease burden, DR-TB, State-transmission model

## Abstract

**Background:**

Currently available injectable agents are inadequate to address the high drug-resistant tuberculosis (DR-TB) burden in China. Regimens including the oral agent bedaquiline have been shown to be efficacious and safe, leading to its incorporation into multiple national TB treatment programs. This analysis evaluated the impact of increased adoption of bedaquiline-containing regimens on the DR-TB burden in China.

**Methods:**

A state-transition model was developed that permits movement and interaction between susceptible, latent, and active TB disease states, while distinguishing between drug-sensitive (DS) and DR-TB. Model inputs were obtained from the published literature or derived such that model metrics approximated those published by the WHO. Expected improvements in infrastructure were built into the model to forecast the epidemiology of DR-TB in China through 2040 in the absence of bedaquiline (baseline forecast). The impact of higher utilization of bedaquiline-containing regimens (85% peak share) was then assessed in two scenarios that differed with regard to treatment success rates of the regimens: 61% (reflecting findings of clinical trials) and 80% (reflecting data from observational studies), versus the 44% success rate associated with standard-of-care treatment.

**Results:**

In the baseline scenario, the model predicted increases in annual incidence of DR-TB by 6–8% during each five-year period between 2020 and 2040, with an increase of 30% over the entire study duration. Adoption of bedaquiline-based regimens limits the incidence increases to only 1–3% in each five-year period and to 8% over the study duration in the 61% success rate scenario. Incidence declines by 1–6% during each five-year period and by 12% over the study duration in the 80% success rate scenario. Similar effects on DR-TB prevalence (4–5% increase in baseline, 0–7% decline in scenario 1, and 4–19% decline in scenario 2) and mortality (5–7% increase in baseline, 0–16% decline in scenario 1, and 6–40% decline in scenario 2) were seen following bedaquiline adoption.

**Conclusions:**

Incorporation of bedaquiline into DR-TB treatment regimens will significantly reduce the DR-TB burden in China, helping to counter the expected increase in burden in the absence of bedaquiline. The study will provide valuable information to public health policy planners.

## Background

According to World Health Organization (WHO) estimates for 2017, China ranked second among the 30 high tuberculosis (TB) burden countries globally, with 889,000 new cases (approximately 9% of the global total) and 38,800 TB-attributable deaths being reported during the year [1]. To tackle the high disease burden on a priority basis, the Chinese government introduced the Directly Observed Treatment, Short Course (DOTS) strategy in 13 provinces that encompassed 50% of the Chinese population in 1991, expanding it nationwide in 2005 [2, 3]. These efforts enabled China to meet the Millennium Development Goal (MDG) of a reduction in TB-related morbidity and mortality by 50% by 2015 [4].

The progress made in reducing the disease burden in China is tempered by the high burden of drug-resistant (DR) TB, including both rifampicin-resistant (RR) and multidrug-resistant (MDR) TB (the latter being defined as resistance to both first-line agents namely isoniazid and rifampicin) [5]. The WHO estimated that 73,000 new cases of DR-TB were reported in China in 2017, with 7.1% of all new cases, 24% of all previously treated cases and 8.2% of overall cases being drug resistant [1]. The government has committed to tackling this disease head-on through its programmatic treatment and management of MDR-TB and XDR-TB patients (PMDRT), employing a multi-frontal attack on DR-TB, centered on improved diagnosis and treatment. However, given the available treatment choices, the overall treatment success rate for DR-TB was reported to be less than 50% in 2015 [6]; this results in continued propagation of the transmission cycle by the treatment-failed patients as they infect the susceptible population.

Projections suggest that the incidence, prevalence, and deaths attributable in China to MDR-TB by 2050 will increase by 62%, 50%, and 53%, respectively, compared with estimates for 2015 [7].

This current and prospective significant burden of DR-TB warrants immediate steps to improve treatment outcomes. Bedaquiline, the first therapeutic agent approved for the treatment of TB in four decades, is an oral agent that was introduced in 2012 and has since been adopted by government-supported DR-TB treatment programs in more than a hundred countries [8, 9]. Its efficacy and tolerability have been amply demonstrated by multiple clinical trials and post-licensure studies that included sizeable patient populations affected by DR-TB [10–17]. Higher bactericidal activity of bedaquiline is expected to make patients less infectious to transmit the disease during treatment [18]. Its higher treatment success rate will mean fewer treatment-failed patients leading to reduction in disease transmission. All of this is expected to mute the transmission cycle, and reduce the spread of DR-TB. Introduction of bedaquiline has been shown to avert disability-adjusted life years [19].

The WHO DR-TB treatment guidelines updated in 2018 place bedaquiline in the category of core first choice drugs, recommending that it be a part of longer regimens for adult patients and children between 6 and 17 years of age [20]. A strategy of providing immediate access to bedaquiline rather than deferring access until failure of first-line treatments has been suggested to be beneficial to both patients and society [21].

This study was carried out to evaluate the long-term impact of increased adoption of bedaquiline-containing regimens on the DR-TB burden in China, with the broader goal of generating the information needed by governmental public health policy decision-makers, non-governmental organizations, and donor entities to make informed decisions regarding bedaquiline incorporation in the national DR-TB treatment program in China.

## Methods

### Model structure

**Fig. 1:**
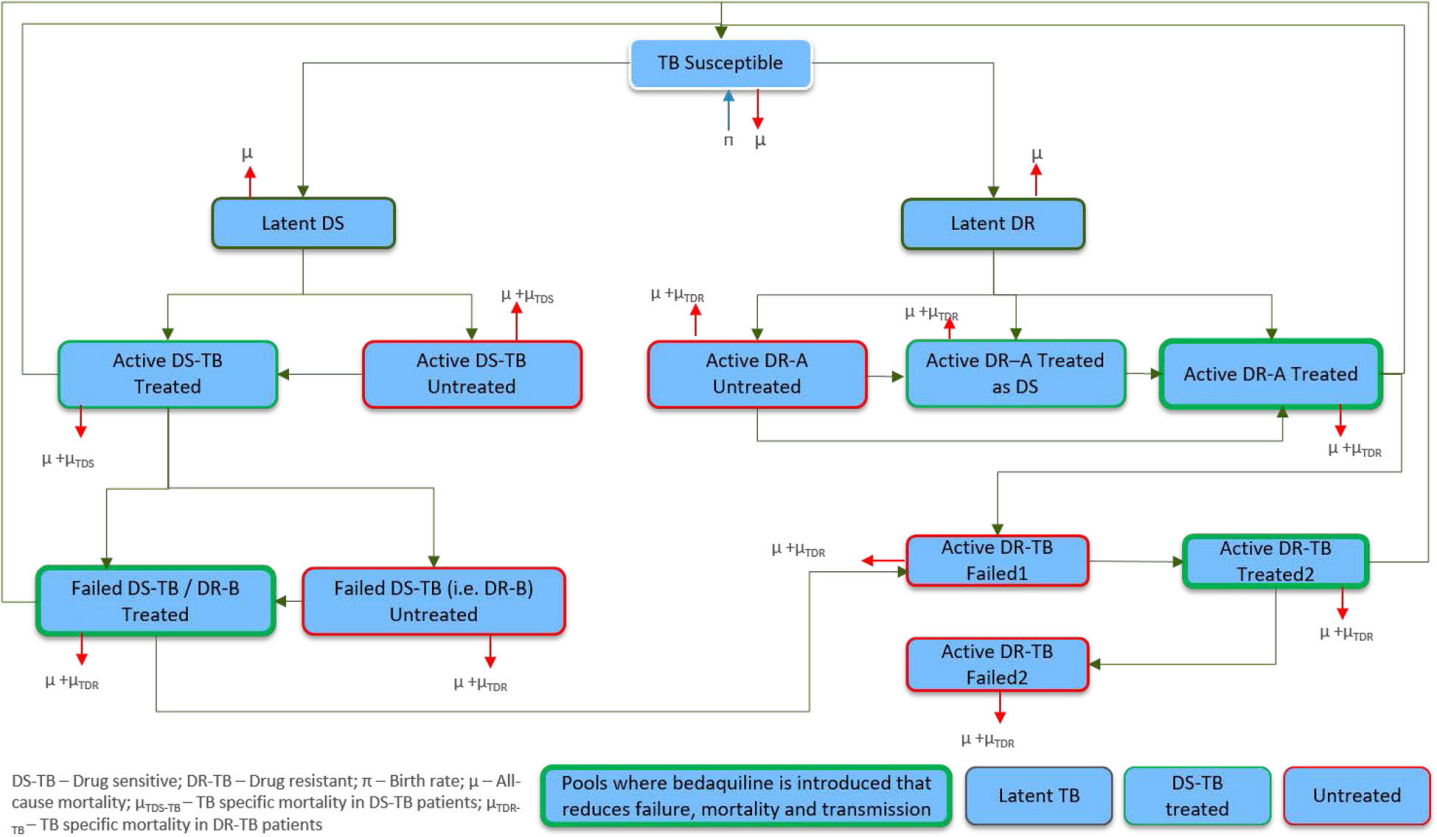
Model structure

The state-transition model developed distinguishes between and considers interaction among three disease states (susceptible, latently infected, and actively infected) and between patients with drug-sensitive (DS) and DR-TB (Fig. 1). The disease is transmitted by patients with active TB to susceptible individuals, who are then considered to be latently infected. Rates of transmission are different for the sputum smear-positive (SS+) and smear-negative (SS−) disease states. Latently infected patients could progress to active TB within a year or slowly during their lifetime [22]. Drug-resistant TB could either result from transmission from active DR-TB patients to susceptible individuals, in which case it was categorized as primary DR-TB, or through DS-TB failing treatment, this category being called acquired DR-TB.

### Model inputs

The published literature and official TB statistics provided several inputs (Additional file 1: Table S1), with additional inputs not obtainable from these sources being deduced so that epidemiological measures resulting from the model were in line with published data available in the reports put forth by the WHO (Table 1).

**Table 1:** Validation of model results with WHO-published data on China between 2015 and 2017

Parameter	Values						External source	Remarks
	Values output in model			Values reported by external sources				
	2015	2017	% change	2015	2017	% change		
Pulmonary TB prevalence	1125 k	1029 k	−8.5%	1119 k	1035 k	−7.5%	WHO Western Pacific Region [23]	See Footnote A
Pulmonary DS-TB incidence	801 k	782 k	−2.4%	813 k	777 k	−4.3%	WHO Global Health Observatory Data Repository [24]	See Footnote B
Pulmonary DR-TB incidence	66 k (2016)	67 k	1.5% (2016–17)	70 k (2016)	70 k	−0.2% (2016–17)		See Footnote C
Pulmonary TB-associated mortality	48 k	46 k	−3.2%	37 k	35 k	−5.7%		See Footnote D

**Footnote A:** Prevalence rate in 2010 was reported to be 108 per 100,000 population, with a CAGR of 4.7% (http://www.wpro.who.int/china/mediacentre/factsheets/tuberculosis/en/). The analysis assumed the CAGR to be the same until 2017. The incidence rate was obtained from WHO (39). Absolute prevalence figures were calculated based on absolute incidence, and the ratio of prevalence per 100 k population to incidence rate.

**Footnote B:** Incidence data reported by WHO (https://www.who.int/tb/publications/global_report/en/) includes non-pulmonary cases. However, ~ 95% of notified TB cases are known to be pulmonary TB cases as seen in the country profile for China in the WHO Global TB report 2018 [1]. Annual pulmonary TB rates from the notified incidence have been applied to total incidence. Additionally, DR-TB incidence has been deducted from total incidence to arrive at the DS-TB incidence.

**Footnote C:** 73 k incident DR-TB cases were estimated for 2017. Of these, 95% were considered to be pulmonary cases (since ~ 95% of notified cases are pulmonary TB cases as seen in the country profile for China in the WHO Global TB report 2018 [1]). The model replicates the WHO-suggested trend of incidence that went up between 2015 and 2016, falling in 2017.

**Footnote D:** Mortality data for only pulmonary cases is not available. To account for this, total TB mortality that included extra-pulmonary cases was used and the share of pulmonary cases applied.

**Abbreviations:** *CAGR* compound annual growth rate; *DR-TB* drug-resistant tuberculosis; *DS-TB* drug-sensitive tuberculosis; *k* thousands; *TB* tuberculosis; *WHO* World Health Organization

Data on birth and all-cause mortality were obtained from the World Bank [25], while future forecasts of these parameters and the population in 2000 (base year) were obtained from the Population Division of the United Nations [26]. China-specific information on notification rates, treatment success rates, and mortality rates associated with DS-TB and DR-TB treatments was obtained from the WHO Global Health Observatory data repository [24].

### Validation of model results

The model closely reflects the current situation with regard to DR-TB in China between 2015 and 2017, as evidenced by the broad overall agreement between the key epidemiological parameters contained in the model and data in the WHO Global Health Observatory Data Repository [24] (Table 1). The published data estimates numbers of prevalent cases, incident cases, and deaths attributable to pulmonary TB to be 1119 thousand (k) cases, 883 k cases, and 35 k deaths respectively in 2015 (after accounting for pulmonary TB), which are in agreement with model results. Incidence figures for pulmonary TB in 2017 are comparable between the model (67 k) and external publications (70 k, which is 95% of the estimated 73 k cases of incident DR-TB reported by the WHO for 2017). The trends of decline between 2015 and 2017 among incidence, prevalence and mortality are also comparable between published sources and the model.

### Modelled strategies and scenario analysis

A baseline forecast scenario between 2020 and 2040 was created not involving the use of bedaquiline. Historical time-series data have documented increased treatment success rates and fewer deaths over time [24], due in part to advances in diagnosis rates and treatment adherence. These trends were assumed to continue in the future (Table 2), with inputs for years between 2020 and 2040 reflecting the epidemiological impact of these advances.

**Table 2:** Anticipated estimates for DR-TB-related incidence, prevalence and mortality changes over time in the baseline (no-bedaquiline scenario)

Year	DR-TB epidemiological parameter (all figures in thousands)		
	Incidence	Prevalence	Mortality
2020	69.9	218.9	30.6
2030	80.7	240.0	34.9
2040	91.0	260.7	38.6

Starting with the baseline scenario, the potential effect of bedaquiline on the epidemiological DR-TB burden in China was assessed in two additional scenarios (Table 3) that captured the different treatment success rates documented both in clinical trials (61%, which was the sputum culture conversion rate at 120 weeks in the bedaquiline arm of clinical trial NCT00449644 [27]) and in post-licensure studies (80%, which reflected the real-world rates reported by the sources listed in Table 4). The post-licensure studies, which documented treatment success rates as high as 93%, were conducted in both developed and developing country settings and included patients with multidrug (MDR) and extensively drug resistant (XDR) TB with more severe disease than DR-TB patients (e.g., additional resistance to fluoroquinolones and involvement of both lungs).

**Table 3:** Bedaquiline success rate and market penetration/adoption inputs utilized for scenario analysis

Parameter		Scenario 1		Scenario 2		
Success rate of first line (LOT1) treatment of DR-TB with bedaquiline		61%		80%		
Peak utilization of bedaquiline-containing regimens in DR-TB treated patients	LOT1	85%		85%		
	LOT2^a^	17%		28%		
Treatment start date		2020-Q1				
		**2020**	**2021**	**2022**	**2023**	**2024**
Adoption of bedaquiline-containing regimens in DR-TB patients (treated with short or long course regimen)		60%	70%	80%	85%	85%

^a^Patients failing LOT1 treatment with bedaquiline were assumed to be ineligible for re-treatment with bedaquiline in LOT2, while 85% of patients not treated with bedaquiline in LOT1 were assumed to be treated with bedaquiline in LOT2 in both scenarios

**Abbreviations:** *LOT* line of treatment; *DR-TB* drug resistant tuberculosis; *Q* quarter of the year

**Table 4:** Real-world bedaquiline treatment success rates reported by post-licensure studies

Study	Year of Publication	Study Type	Country	Number of Patients	Study Population	Comparator	Efficacy (Success Rate)	
							BDQ	SOC
WHO [28]	2017	Systematic literature review conducted in 2016	Multi country	205	MDR and XDR	Not mentioned	61.0%	–
			France	45		Not mentioned	75.5%	–
			South Africa	195		Not mentioned	63.4%	–
Borisov et al. [12]	2017	Large retrospective, observational study conducted in 25 centers in 15 countries on 5 continents	Africa	113	MDR and XDR	Not mentioned	64.6%	–
			Eastern Europe	85		Not mentioned	63.5%	–
			Other settings	49		Not mentioned	55.1%	–
Skrahina et al. [11]	2018	Study on 192 MDR-TB patients treated with bedaquiline	Belarus	192	MDR	Not mentioned	92.7%	–
Diacon et al. [10]	2014	Phase 2b trial	Brazil, India, Latvia, Peru, the Philippines, and Russia	132 (66 each)	MDR	Standard-of-care drugs	62.0%	44.0%
							58.0%	32.0%
Guglielmetti et al. [14]	2017	Retrospective study of multicenter observational cohort	France	45	MDR and XDR	Not mentioned	80%	–
Ndjeka et al. [15]	2018	Retrospective study	South Africa	200	MDR and XDR	Only bedaquiline	69.5%	–

**Abbreviations:** *WHO* World Health Organization; *MDR-TB* drug-resistant tuberculosis; *XDR-TB* extensively drug-resistant tuberculosis

The peak annual share of bedaquiline-containing regimens for first-line (LOT1) treatment, set at 85%, was deemed to be attainable by 2024 (Table 3). Of this, 10% was considered to be through short-course regimens. Patients failing LOT1 treatment with bedaquiline were assumed to be ineligible for re-treatment with bedaquiline in LOT2, while 85% of patients not treated with bedaquiline in LOT1 were assumed to be treated with bedaquiline in LOT2 in both scenarios.

## Results

### Baseline scenario

**Fig. 2:**
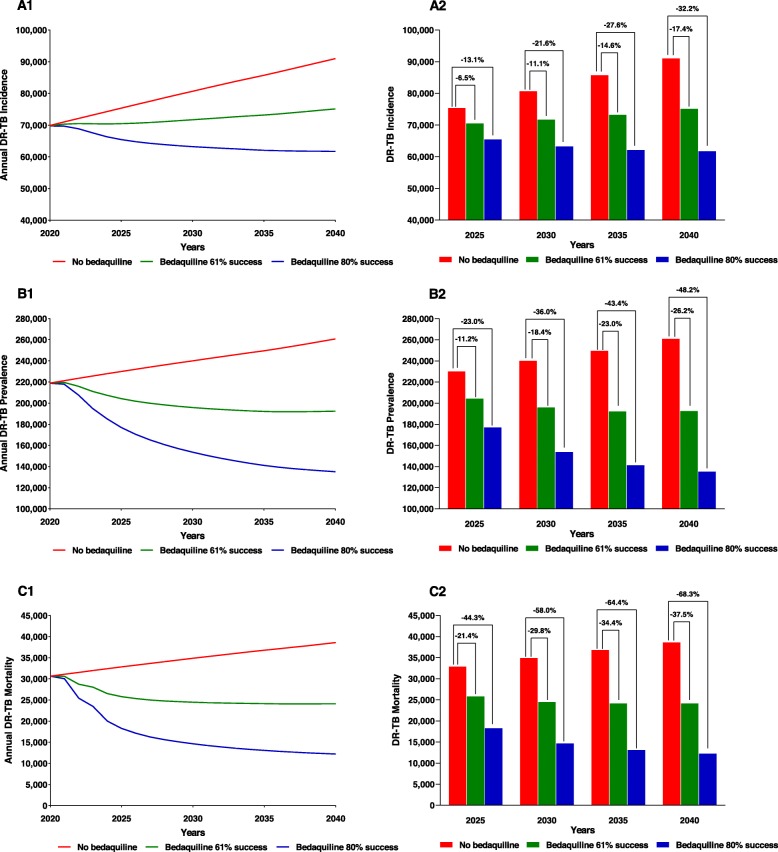
Trends and reductions in DR-TB incidence (A1 and A2), prevalence (B1 and B2), and mortality (C1 and C2) as a result of a theoretical expansion of bedaquiline treatment for DR-TB under 2 treatment success rate scenarios

The model predicts that not using bedaquiline for the treatment of DR-TB results in increases in the rates of all three epidemiological parameters between 2020 and 2030 as well as between 2030 and 2040 (Table 2 and Fig. 2). Incidence was predicted to increase by 15.4% and 12.8%, respectively, during the two consecutive decades. Corresponding increases in prevalence were predicted to be by 9.6% and 8.6%, respectively, while mortality was predicted to increase correspondingly by 14.0% and 10.6%, respectively.

### Scenarios evaluated based on different success rates assumed for bedaquiline

In the first scenario of bedaquiline utilization (61% treatment success rate), incidence was predicted to increase at significantly lower rates than in the baseline scenario, whether viewed in five-year intervals or over the entire duration of the analysis (Table 5 and Fig. 2). Rates of increase in incidence during the five-year intervals 2020–2025, 2025–2030, 2030–2035, and 2035–2040 were predicted to be 1%, 2%, 2%, and 3%, respectively, considerably lower than the corresponding increases predicted in the baseline forecast. Over the 2020–2040 period, as compared to an increase in incidence of 30% in the baseline forecast, use of bedaquiline showed an increase of only 8%.

**Table 5:** Effect of bedaquiline use for the treatment of DR-TB on incidence, prevalence and mortality in 5-year increments between 2020 and 2040 in China

Cumulative reduction (%, absolute)		Change in each five-year period				Change over the period 2020–2040
		2020–2025	2025–2030	2030–2035	2035–2040	
Incidence	No bedaquiline	8%	7%	6%	6%	30%
	Bedaquiline use; 61% success rate	1%	2%	2%	3%	8%
	Bedaquiline use; 80% success rate	−6%	−3%	−2%	−1%	−12%
Prevalence	No bedaquiline	5%	4%	4%	4%	19%
	Bedaquiline use; 61% success rate	−7%	−4%	−2%	0%	−12%
	Bedaquiline use; 80% success rate	−19%	−13%	−8%	−4%	−38%
Mortality	No bedaquiline	7%	6%	5%	5%	26%
	Bedaquiline use; 61% success rate	−16%	−5%	−1%	−0%	−21%
	Bedaquiline use; 80% success rate	−40%	−20%	−11%	−6%	−60%

This scenario was expected to lead to a decline of DR-TB prevalence by 7%, 4%, 2%, and 0% respectively, during the corresponding five year periods. This is in contrast to the increases estimated in the baseline scenario. Overall, as compared to the baseline forecast that showed an increase in prevalence of 19% over the 20-year period 2020 to 2040, the scenario involving bedaquiline use resulted in a decrease in prevalence by 12% over this period. During this period, mortality was also predicted to decline by 21%, as compared to an increase of 26% in the baseline forecast.

The second scenario (80% success rate) was notable for even greater declines in all three epidemiological parameters (Table 5 and Fig. 2) compared to the first scenario. This scenario was predicted to decrease the incidence by 6%, 3%, 2% and 1%, respectively, during each successive five-year period, or a cumulative decline of 12% over the 2020–2040 period. Declines in prevalence were also predicted to be greater in magnitude than in the first scenario (declines over baseline of 19%, 13%, 8%, and 4% during the five-year intervals, or decline of 38% over the 20-year period). Similarly, mortality was also predicted to decrease over baseline more appreciably than in the first scenario (declines of 40%, 20%, 11%, and 6% during the successive five-year intervals, or 60% over the 20-year period).

### Summary

In this 20-year prospective cohort review to understand the long-term impact of bedaquiline on DR-TB burden trends in China,

- In the baseline treatment scenario with no breakthrough in curative intervention, an increase in DR-TB incidence, prevalence, and mortality is seen over the forecast period, despite the assumptions made with regard to future enhancements in the treatment infrastructure.
- In the first scenario of increased use of bedaquiline that assumed moderately superior treatment success rate versus SOC and widespread adoption, the increase seen in the baseline analysis in DR-TB incidence becomes muted, while DR-TB prevalence and related deaths shows an actual decline.
- In the second scenario of increased use of bedaquiline, assuming a higher treatment success rate and widespread adoption, the study shows a significant decline over the evaluation period in all three epidemiological metrics – incidence, cases, and deaths.

## Discussion

The current burden of DR-TB in China is sizeable according to recent estimates published by the WHO [1], and threatens to undo the significant progress made in the treatment of TB by the nationwide implementation of the DOTS strategy in 2005 [3]. The burden of DR-TB as it stood in 2017 was estimated to result in future losses to the Chinese GDP to the tune of $1.2 billion [29]. The current analysis shows a 6–8% increase in the future incidence of DR-TB predicted occurring during each five-year period between 2020 and 2040, and overall increase by 30% by 2040 following the current DR-TB treatment scenario. Drug-resistant TB has far reaching consequences, including poor treatment outcomes [30], increased potential for disease transmission by the greater number of patients failing treatment [31], increased likelihood of reinfection of successfully treated patients with resistant strains [32–34], and adverse effects on patients’ health-related quality of life (HRQoL) [35]. The regimens that represent the current standard of care are not capable of addressing the burden of DR-TB, as evidenced by low treatment success rates of less than 50% [6].

A strategy that includes the utilization of a bedaquiline-containing regimen, whose treatment success rates among DR-TB patients has been documented to be as high as 93%, can potentially provide the solution to the possible public health crisis posed by DR-TB in the coming years in China. With its proven efficacy documented in clinical trials [9] and effectiveness reported by post-licensure real-world observational studies [10–12, 14, 15, 28], bedaquiline has the potential to provide a viable solution to the DR-TB challenge faced in China, and thereby supplement the infrastructural improvements being put in place by the government. The overall impact of the intervention will be strongly influenced by the availability of resources, tools, and infrastructure as well as the uptake and adherence in the population.

In 2018, bedaquiline was made available to an initial group of DR-TB patients under a pilot program called the New Drug Introduction and Protection (NDIP) [36], marking an important next step in the renewed fight against DR-TB in China.

This modeling analysis relied on some simplifications and may have limitations. Two assumptions that were made may not fully hold: that the entire population is equally susceptible to infection and that the rate at which bedaquiline is accessible by the population is uniform throughout China. If these assumptions do not hold, the impact of bedaquiline-containing regimens on the DR-TB burden may be different in magnitude from estimates predicted by this analysis, although the directional impact on incidence, prevalence, and mortality is still expected to hold. A second limitation was that the analysis did not examine the potential impact of the availability of upcoming or future treatments, with a view to measure the isolated impact of bedaquiline. Further, the model does not differentiate between TB in pediatric and adult populations, which differ in degree of infectivity, smear status and diagnosis rate.

## Conclusions

Broader use of bedaquiline-containing regimens in China based on current global standards for the treatment of DR-TB has the potential to significantly lower the disease burden and counter the increases in incidence, prevalence, and mortality otherwise expected to ensue with the *status quo*. This analysis is therefore considered to be a critical element in policy discussions on the adoption of new DR-TB regimens that have the potential to bend the trajectory of the current DR-TB crisis in China.

## Supplementary information

**Additional file 1.** Detailed list of inputs for the DR-TB model.

## Data Availability

The datasets supporting the conclusions of this article are contained within the article and its supporting files.

## Abbreviations

CAGR: Compound annual growth rate
DOTS: Directly Observed Treatment, Short Course
DR-TB: Drug-resistant tuberculosis
DS-TB: Drug-sensitive tuberculosis
HRQoL: Health-related quality of life
k: Thousand
MDG: Millennium Development Goal
MDR: Multidrug resistant
QoL: Quality of life
SS −: Sputum smear-negative disease
SS +: Sputum smear-positive disease
TB: Tuberculosis
WHO: World Health Organization
XDR: Extensively drug resistant
