# Supplementary material for: Long-term impact of the adoption of bedaquiline-containing regimens on the burden of drug-resistant tuberculosis in China
Source: BMC Infect Dis. 2020 Feb 10;20:113. doi: 10.1186/s12879-020-4795-4 (PMC7011376; doi:10.1186/s12879-020-4795-4)
Supplement: Supplementary file 1 — Additional file 1. Detailed list of inputs for the DR-TB model. [file 12879_2020_4795_MOESM1_ESM.docx]

**Additional File 1: Table 1**

| **Parameter** | | **Value** | | **Source and definitions** |
| --- | --- | --- | --- | --- |
|  |  | **2016** | **2040** |  |
| **Demographic inputs** | | | | |
| Active DS-TB as a proportion of latent TB population | | 36% in 1999 | | Dye et al. (1999) [1] |
| Relative transmissibility from active DS-TB SS– (relative to SS+) | | 24%  (across years) | | Tostmann et al. (2008) [2]; Lawn et al. (2013) [3] |
| **Active DS-TB** | | | | |
| Proportion of active DS-TB incidence diagnosed | | 87% | | Data from the WHO Global Health Observatory data repository [4] was used to calculate the proportion of patients diagnosed within a quarter of getting active DS-TB disease (74%). This proportion is extrapolated to grow to 90% by 2040 |
| Proportion of diagnosed incident patients initiating treatment | | 100%  (across years) | | All diagnosed and notified patients were considered to initiate treatment |
| Proportion SS+ | | 30% | 30% | The WHO Global Health Observatory data repository [4] reports proportion of SS+ patients to be ~50% among all pulmonary TB patients notified between 2005 and 2010, falling to 30% by the end of 2012, with the same rate being assumed to continue till 2040 |
| **Active DS-TB treated** | | | | |
| Average time to sputum smear conversion (in months) | | 1  (constant across years) | | Kim et al. (2016) [5] |
| Duration of therapy (in months) | | 6  (constant across years) | | CDC [6] |
| % cured (of treatment starts) | | 97% | 97% | Generally acknowledged cure rates for DS-TB in China, whose DOTS program has historically been highly successful [7] |
| **Failed DS-TB (i.e., DR-B)^a^ or DR-A LOT1 treated – comparison of SoC and bedaquiline** | | | | |
| Average time to sputum smear conversion (in months) | SoC^b^ | 3  (constant across years) | | Fortún et al. (2007) [8] |
|  | Bedaquiline^b^ | 2  (constant across years) | | In clinical trials, bedaquiline exhibited faster bactericidal activity at 8 weeks compared to SoC |
| Duration of therapy (in months) | SoC^b^ | 18  (across years) | | Cheepsattayakorn (2013) [9] stated a duration of 18 months for SoC before 2016  The WHO recommended a SCR in 2016, which is approximately 10 months [10]. For China, the SCR was assumed to have a 10% penetration, which amounts to a weighted average of 18 months  In clinical trials, bedaquiline has had the same total course duration as SoC [11] |
|  | Bedaquiline^b^ |  |  |  |
| % succeeded (of treatment starts) | SoC^b^ | 44%  (across years) | | The WHO Global Health Observatory data repository [4] reports rates close to 45% from 2008–2014. A Chinese survey documented rates of 44.6% [12]. These findings are similar to the sputum culture conversion rate observed at 120 weeks in the non-bedaquiline arm of clinical trial NCT00449644 [13] |
|  | Bedaquiline^b^; 61% success rate | 61%  (across years) | | 61% sputum culture conversion rate at 120 weeks seen in the bedaquiline arm of clinical trial NCT00449644 [13] |
|  | Bedaquiline^b^; 80% success rate | 80%  (across years) | | Higher side sputum culture conversion rate scenario considered for assessment of bedaquiline impact in this analysis |
| % deaths during treatment (of treatment starts) | SoC^b^ | 20%  (across years) | | According to a retrospective analysis in China [14], 52% of patients failed treatment and 9% of the 52% died during treatment. Of the patients lost to follow up, it is assumed that a larger proportion would have died by the end of the treatment tenure. As a result, 20% is the assumed total treatment death rate as opposed to the 9% of the proportional rate of follow up of 74% (=12%) |
|  | Bedaquiline^b^; 61% success rate | 14%  (across years) | | 39% and 56% were the failure rates in the bedaquiline and non-bedaquiline arms, respectively, at 120 weeks in the clinical trial NCT00449644 [13]; this reduction in treatment failure rate with bedaquiline (based on its superior success rate) has been applied in the same proportion to calculate the death rate in the two bedaquiline adoption scenarios |
|  | Bedaquiline^b^; 80% success rate | 7%  (across years) | |  |
| **Active DR-A** | | | | |
| % active DR-A correctly diagnosed as DR-A | | 23% | 98% | WHO Global Health Observatory data repository [15], assumed to improve to 98% correct diagnosis before 2040, same as for DS-TB |
| % active DR-A misdiagnosed and treated as DS | | 51% | 0% | Taken to be total diagnosis rate less those correctly diagnosed as DR-TB (above row) |
| % active DR-A diagnosed patients initiating treatment | | 100%  (across years) | | Diagnosis rates taken to be the notifications rate and all notified patients were assumed to initiate treatment |
| % SS+ | | 30% | 30% | Same as DS-TB |
| **DR-TB LOT2 treated – comparison of SoC and bedaquiline** | | | | |
| Average time to sputum smear conversion  (in months) | SoC^b^ | 3  (across years) | | Fortún et al. (2007) [8] |
|  | Bedaquiline^b^ | 2  (across years) | | Bedaquiline exhibits faster bactericidal activity at 8 weeks compared to SOC in clinical trials [13] |
| Duration of therapy  (in months) | SoC^b^ | 21  (across years) | | Cheepsattayakorn (2013) [9] reports the 21-month duration  The Nix-TB trial [16] targeting XDR-Tb patients aims to cure patients in 6-9 months. Half of all XDR-TB treatment courses in China are assumed to be short course treatments starting in 2020, making the weighted average duration approximately 15 months  In bedaquiline clinical trials [11], the total course duration for bedaquiline was the same as for the SoC |
|  | Bedaquiline^b^ | 21  (across years) | |  |
| % cured  (of treatment starts) | SoC^b^ | 32%  (across years) | | WHO Global Health Observatory data repository [4] |
|  | Bedaquiline^b^; 61% success rate | 45%  (across years) | | 61% and 44% were the sputum culture conversion rates in the bedaquiline and non-bedaquiline arms, respectively, at 120 weeks in the NCT00449644 trial [13], translating to bedaquiline’s 39% superior efficacy; this 39% superiority has been applied to the cure rate taken for SoC each year |
|  | Bedaquiline^b^; 80% success rate | 58%  (across years) | |  |
| % deaths during treatment (of treatment starts) | SoC^b^ | 33%  (across years) | | Shah et al. (2008) [17] report that XDR-TB has a 5:3 mortality rate compared to DR-TB under similar supervision. The same ratio was therefore applied to the DR-TB treatment-associated mortality in China. |
|  | Bedaquiline^b^; 61% success rate | 27%  (across years) | | 39% and 56% were the treatment failure rates in the bedaquiline and non-bedaquiline arms, respectively, at 120 weeks in the NCT00449644 trial [13]; this 30% reduction in failure rate seen with bedaquiline (based on its superior success rate) has been applied in the same proportion to calculate the death rate in the two bedaquiline adoption scenarios |
|  | Bedaquiline^b^; 80% success rate | 20%  (across years) | |  |

^a^DR-B patients are DS-TB patients acquiring DR-TB status through failure of treatment, while DR-A patients are susceptible patients who got infected directly from an active DR-TB patient

^b^SoC regimen refers to injection-based regimens without bedaquiline, while bedaquiline refers to non-injection based oral regimens that include bedaquiline

**Abbreviations:** CDC, United States Centers for Disease Control and Prevention; DOTS, Directly observed treatment, short-course; DR-TB, drug-resistant tuberculosis; DS-TB, drug-sensitive tuberculosis; LOT, line of treatment; SCR, short course regimen; SoC, standard of care; SS−, sputum smear-negative; SS+, sputum smear-positive; TB, tuberculosis; WHO, World Health Organization;

**References**

1. Dye C, Scheele S, Dolin P, Pathania V, Raviglione MC: **Consensus statement. Global burden of tuberculosis: estimated incidence, prevalence, and mortality by country. WHO Global Surveillance and Monitoring Project**. *Jama* 1999, **282**(7):677-686.

2. Tostmann A, Kik SV, Kalisvaart NA, Sebek MM, Verver S, Boeree MJ, van Soolingen D: **Tuberculosis transmission by patients with smear-negative pulmonary tuberculosis in a large cohort in the Netherlands**. *Clinical infectious diseases : an official publication of the Infectious Diseases Society of America* 2008, **47**(9):1135-1142.

3. Lawn SD, Edwards D, Wood R: **Tuberculosis transmission from patients with smear-negative pulmonary tuberculosis in sub-Saharan Africa**. *Clinical infectious diseases : an official publication of the Infectious Diseases Society of America* 2009, **48**(4):496-497.

4. **Global Health Observatory data repository: Tuberculosis** [<http://apps.who.int/gho/data/node.main.1315?lang=en>]

5. Kim J, Kwak N, Lee HY, Kim TS, Kim CK, Han SK, Yim JJ: **Effect of drug resistance on negative conversion of sputum culture in patients with pulmonary tuberculosis**. *International journal of infectious diseases : IJID : official publication of the International Society for Infectious Diseases* 2016, **42**:64-68.

6. **TB Elimination: The Difference Between Latent TB Infection and TB Disease** [<https://www.cdc.gov/tb/publications/factsheets/general/LTBIandActiveTB.pdf>]

7. Kanabus A: **TBFacts.org: TB in China - TB control, DOTS, MDR-TB**. In*.* Horsham, West Sussex, England: Global Health Education; 2019.

8. Fortún J, Martin-Davila P, Molina A, Navas E, Hermida JM, Cobo J, Gomez-Mampaso E, Moreno S: **Sputum conversion among patients with pulmonary tuberculosis: are there implications for removal of respiratory isolation?** *The Journal of antimicrobial chemotherapy* 2007, **59**(4):794-798.

9. Cheepsattayakorn A: **Drug-Resistant Tuberculosis – Diagnosis, Treatment, Management and Control: The Experience in Thailand**. In: *Tuberculosis - Current Issues in Diagnosis and Management.* Edited by Mahboub HB, Vats MG. London, UK: Intech Open; 2013: 26 pages.

10. World Health Organization: **The Shorter MDR-TB Regimen**. In*.*, vol. 2019. Geneva, Switzerland: World Health Organization; 2016.

11. **Sirturo^®^ - Clinical Trials** [<https://www.sirturo.com/sirturo-clinical-trials.html>]

12. Xu C, Pang Y, Li R, Ruan Y, Wang L, Chen M, Zhang HJJoI: **Clinical outcome of multidrug-resistant tuberculosis patients receiving standardized second-line treatment regimen in China**. 2018, **76**(4):348-353.

13. Diacon AH, Pym A, Grobusch M, Patientia R, Rustomjee R, Page-Shipp L, Pistorius C, Krause R, Bogoshi M, Churchyard G *et al*: **The diarylquinoline TMC207 for multidrug-resistant tuberculosis**. *The New England journal of medicine* 2009, **360**(23):2397-2405.

14. Xu C, Pang Y, Li R, Ruan Y, Wang L, Chen M, Zhang H: **Clinical outcome of multidrug-resistant tuberculosis patients receiving standardized second-line treatment regimen in China**. *J Infect* 2018, **76**(4):348-353.

15. **Global Health Observatory data repository** [<http://apps.who.int/gho/data/view.main.57200>]

16. TB Alliance: **Nix-TB: Testing a New Potential Treatment for XDR-TB**. In*.*: TB Alliance; 2018.

17. Shah NS, Pratt R, Armstrong L, Robison V, Castro KG, Cegielski JP: **Extensively drug-resistant tuberculosis in the United States, 1993-2007**. *Jama* 2008, **300**(18):2153-2160.
